# Synovial tissue volume: a treatment target in knee osteoarthritis (OA)

**DOI:** 10.1136/annrheumdis-2014-206927

**Published:** 2015-06-26

**Authors:** Terence W O'Neill, Matthew J Parkes, Nasimah Maricar, Elizabeth J Marjanovic, Richard Hodgson, Andrew D Gait, Timothy F Cootes, Charles E Hutchinson, David T Felson

**Affiliations:** 1 Faculty of Medical and Human Sciences, Arthritis Research UK Centre for Epidemiology, Institute of Inflammation and Repair, Manchester Academic Health Science Centre, University of Manchester, Manchester, UK; 2 NIHR Manchester Musculoskeletal Biomedical Research Unit, Central Manchester NHS Foundation Trust, Manchester Academic Health Science Centre, Manchester, UK; 3 Department of Rheumatology, Salford Royal NHS Foundation Trust, Salford, UK; 4 Centre of Imaging Sciences, Institute of Population Health, University of Manchester, Manchester, UK; 5 UK Warwick Medical School, The University of Warwick, Coventry, UK; 6 Clinical Epidemiology Unit, Boston University School of Medicine, Boston, Massachusetts, USA

**Keywords:** Knee Osteoarthritis, Synovitis, Inflammation

## Abstract

**Background:**

Synovitis occurring frequently in osteoarthritis (OA) may be a targeted outcome. There are no data examining whether synovitis changes following intra-articular intervention.

**Methods:**

Persons aged 40 years and older with painful knee OA participated in an open label trial of intra-articular steroid therapy. At all time points they completed the Knee Injury and Osteoarthritis Outcome Score (KOOS) questionnaire. They had a contrast-enhanced (CE) MRI immediately prior to an intra-articular steroid injection with a repeat scan within 20 days. Response status was assessed using the Osteoarthritis Research Society International (OARSI) response criteria. OARSI responders were followed until their pain relapsed either within 20% of baseline or 6 months, shortly after which a third MRI was performed. Synovial tissue volume (STV) was measured on postcontrast knee images. We looked at changes in the STV and in pain, and their association.

**Results:**

120 subjects with preinjection and postinjection CE MRI were followed. Their mean age was 62.3 years (SD=10.3) and 62 (52%) were women. The median time between injection and follow-up scan was 8 days (IQR 7–14 days). 85/120 (71%) were OARSI responders. Pain decreased (mean change in KOOS=+23.9; 95% CI 20.1 to 27.8, p<0.001) following steroid injection, as did mean STV (mean change=−1071 mm^3^; 95% CI −1839 mm^3^ to −303 mm^3^, p=0.01). Of the 80 who returned for a third MRI, pain relapsed in 57, and in the 48 of those with MRI data, STV increased between follow-up and final visit (+1220 mm^3^; 95% CI 25 mm^3^ to 2414 mm^3^, p=0.05). 23 were persistent responders at 6 months and, in these, STV did not increase (mean change=−202 mm^3^; 95% CI −2008 mm^3^ to 1604 mm^3^, p=0.83). Controlling for variation over time, there was a significant association between synovitis volume and KOOS pain (b coefficient—change in KOOS pain score per 1000 mm^3^ change in STV=−1.13; 95% CI −1.87 to −0.39, p=0.003), although STV accounted for only a small proportion of the variance in change in pain.

**Conclusions:**

Synovial tissue volume in knee OA shrinks following steroid therapy, and rebounds in those whose pain relapses. It can be considered a treatment target in symptomatic knee OA.

**Trial registration number:**

ISRCTN07329370.

## Introduction

Synovitis is a well-recognised feature of knee osteoarthritis (OA). On arthroscopy, synovitis is seen in approximately 50% of the knees of patients with painful OA and in an even higher percentage using MRI.^1^ ^2^ Loeuille *et al*^3^ comparing MRI, histologic and arthroscopic appearance of synovium in persons with symptomatic knee OA reported a high correlation between the degree of synovial thickening on MRI and macroscopic scoring of synovitis by an arthroscopist (r=0.58). Thickness was also correlated with infiltration of inflammatory cells into the subsurface layers of synovium (r=0.46). Thus, synovial thickening observed from gadolinium-enhanced MRI correlates with macroscopic and microscopic evidence of synovitis and is typically referred to as synovitis.

Using non-contrast enhanced MRI, Hill and colleagues reported that synovitis was correlated cross-sectionally with the severity of knee pain in persons with knee OA.^4^ Hill also found a modest correlation (r=0.21) between change in synovitis on MRI with change in severity of knee pain over time in 270 persons with symptomatic knee OA who had undergone serial MRIs.^5^ These findings have been corroborated by Zhang and colleagues using data from serial MRIs in the Multicenter Osteoarthritis Study (MOST).^6^ However, the results from these studies are limited by the use of non-contrast enhanced MRI to characterise synovitis. Using a non-contrast technique, it is difficult to distinguish synovitis from effusion, and some areas of synovitis are impossible to differentiate from surrounding structures.^2^ ^7^ ^8^ Contrast enhances the appearance of synovium without showing the other surrounding structures, and its use is critical to best identify synovitis. Baker *et al*^9^ reported on a subset of the MOST cohort who had gadolinium-enhanced MRIs and found that the contrast-enhanced synovitis appeared to be strongly associated with pain. Synovitis was far more prevalent in the knee pain group.

Intra-articular corticosteroids have long been a mainstay of OA treatment, thought to act through their anti-inflammatory effect. In a meta-analysis of randomised trials comparing corticosteroid with placebo injections for the treatment of knee OA, Arroll and Goodyear-Smith^10^ reported the superiority of corticosteroid injections. The treatment effect was large, and the number needed to treat ranged from 1.3 to 3.5. In meta-analyses, steroids are more effective than placebo injection for up to 2 weeks after steroid injection, but, for many patients, the beneficial effect is much longer.^10^ In rheumatoid arthritis, a reduction in synovial volume has been seen after intra-articular steroid injections, but the effect of treatment on synovitis in OA has not, to our knowledge, been studied.^11^

Given the interest in testing OA treatments targeting synovial inflammation, knowledge of whether measurable reduction in synovitis occurs with appropriate treatment is needed. Such data are important to serve as evidence that synovial volume changes in response to intervention, and would be a key milestone in considering synovial volume as an outcome measure in clinical trials of knee OA. Also, if synovitis diminishes, it is unclear whether this is correlated with pain reduction. Further, there is no evidence of longer term effects of intra-articular steroids on structure and particularly whether synovitis rebounds after treatment.

The aims of this study were to determine (i) whether synovial tissue volume (STV) as assessed using contrast-enhanced MRI changes in response to intra-articular steroid therapy and (ii) whether change in symptoms of pain correlates with changes in STV. Our study design, in which we examined MRIs before and after steroid injection and also obtained a third MRI on relapse of pain, allowed us also to examine the relation of pain fluctuation to change in STV.

## Methods

### Subjects

Men and women aged 40 years and over were recruited from both primary and secondary care clinics for participation in an open label study to observe the efficacy of intra-articular steroid therapy in symptomatic knee OA (ISRCTN: 07329370). Subjects were included if they reported moderate knee pain for more than 48 h in the previous 2 weeks or scored greater than 7 out of 32 on the Knee Injury and Osteoarthritis Outcome Score (KOOS) questionnaire, questions P2–P9 (Question P1 relates to frequency of knee pain, which is irrelevant given the inclusion criteria on pain frequency). Inclusion criteria included imaging confirmation of OA either radiologically (in any joint on anterio-posterior (AP), skyline or lateral knee radiographs obtained within the previous 2 years) with a Kellgren-Lawrence score of two or more or, on MRI scan or at arthroscopy. For MRI and arthroscopy, we required typical changes of OA with at least cartilage loss present. Exclusion criteria included the presence of secondary OA from gout, previous septic arthritis or inflammatory arthritis, injection with hyaluronic acid or steroid injection within the previous 3 months, history of knee surgery within the previous 6 months, concurrent life-threatening illness and any contraindication to MRI scanning. Subjects were provided with an information sheet about the study and those who agreed to take part subsequently provided written informed consent.

### Screening and baseline assessment

Those who were interested initially attended a screening visit to determine eligibility. Where subjects had not had a knee radiograph in the previous 2 years, or other imaging evidence of OA, knee radiographs were performed. Blood was also taken to assess renal function. Those with an estimated glomerular filtration rate less than 40 mL/min were subsequently excluded from the study. Those who were eligible and fulfilled the inclusion/exclusion criteria were invited to attend a baseline visit. Subjects also completed a series of questionnaires including the KOOS pain scale and a visual analogue scale (VAS) score for pain during an activity that a patient nominated as being most troublesome (VAS_NA_).^12^ Subjects had a CE MRI scan performed with gadolinium as the contrast agent followed by an intra-articular steroid injection within a couple of hours (see below).

### Intervention

Arthrocentesis was performed using an 18G needle by one of two experienced clinicians (TWON/NM) using a medial approach to the knee joint. Any synovial fluid obtained was forwarded for synovial fluid analysis. Using the same needle, the knee was then injected with 80 mg methylprednisolone (without local anaesthetic). Any subject in whom the synovial fluid white cell count (WCC) was found to be greater than 1.5×10^9^/L was subsequently withdrawn from the study because of a concern that they may not have OA.

### Follow-up

**Figure 1:**
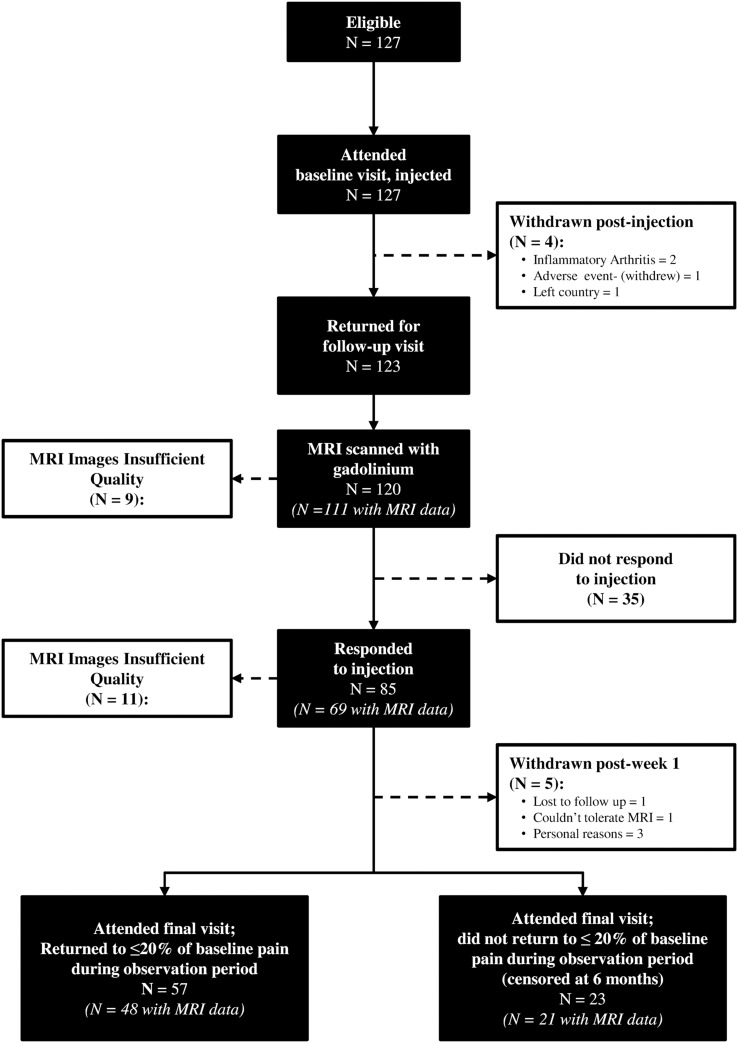
Study flow diagram.

We endeavoured to see all subjects within 14 days of injection. They completed the KOOS and VAS scores and had a repeat CE MRI. Response was assessed using the Outcome Measures in Rheumatology Clinical trials (OARSI-OMERACT) responder criteria using the KOOS pain scale to determine responder status.^13^ A responder was defined as either (i) a greater or equal to 20% change in KOOS pain score, with an absolute change of at least 3 units if the baseline score was 15 or less, and a global improvement in pain using a 5 category variable, or (ii) a greater or equal to a 50% change in the KOOS pain score with an absolute change of at least 3 units if the baseline score was 15 or less. Those who did not respond were not followed up. Those who responded were followed up by regular telephone calls during which the same KOOS survey questions were administered. Those whose pain recurred to within 20% (of the baseline KOOS pain subscale score) were defined as having relapsed and were invited for a final MRI. Those whose pain levels did not return to this level at 6 months of follow-up were classified as having ‘persistently responded’ and had a final MRI scan scheduled at which point the study ended (see figure 1).

### Magnetic resonance imaging: acquisition and analysis

**Figure 2:**
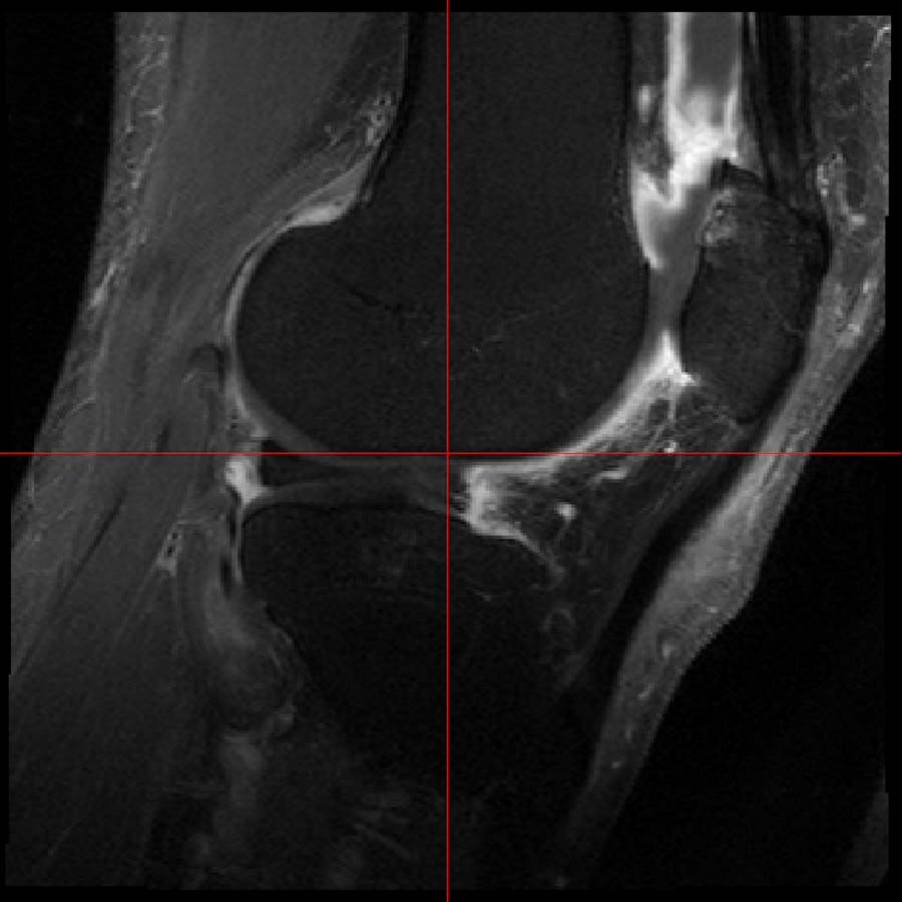
Gadolinium-enhanced MRI of study patient, showing synovial tissue and fluid differentiation.

Using a 3T Philips MRI, we obtained sagittal postcontrast T1W FS (TR 550 ms, TE 20 ms, FOV 14 cm×14 cm, size 320×320) and sagittal precontrast 3D WATSc (TR 20 ms, TE4.7 ms, FOV 15 cm×15 cm, size 288×288) scans in all subjects at baseline, within 2 weeks of follow-up and at a third, final visit, for patients whose symptoms recurred within 6 months or if they had not recurred at 6 months. An example of a postcontrast sagittal image from the study is shown in figure 2. Manual segmentation of the synovial tissue layer was performed on the sagittal postcontrast T1W FS image by a single observer. To optimise the ability to detect changes in synovial volume, segmentations were carried out paired though blinded to order—repeated MRIs of a specific knee were segmented before moving to the next knee, with the visit order randomised by a separate member of the research team who took no part in the segmentation. Using computer image analysis, we excluded the cartilage within the segmented space by thresholding in the associated sagittal (3D WATSc: TR 20 ms, TE 7.7 ms, FoV 16 cm, 288×288) scan. The rest of the segmented space was assumed to be a mixture of fluid and synovial tissue. We calculated the proportion of synovial tissue in every voxel using P=(I−m_f_)/(m_s_−m_f_) truncated to [0, 1], where I is the voxel intensity, and m_f_ and m_s_ are the means of the intensity distributions of fluid and STV, respectively. To evaluate repeatability of synovial volumes, the segmenter was asked to segment 10 knees randomly selected (without replacement) from those that had been previously segmented with new identification numbers that were assigned. In addition, a sample of 101 patients’ images (262 images in total, across the three study visits) were evaluated semi-quantitatively for the presence of synovitis by an experienced radiologist using an approach previously described,^14^ which comprised scoring each of 11 areas from 0–1–2 in increasing order of severity, with a resulting total score up to 22.

### Statistical analysis

Subject characteristics and STVs were described with means and SDs for normally distributed variables and medians and interquartile ranges for variables with a skewed distribution. We assessed intraobserver repeatability of the manual segmentation process using intraclass correlation (ICC). The association between quantitative and semiquantitative approaches to assessing STV was evaluated using Spearman's correlation coefficient.

The primary outcome was to assess change in knee pain from baseline to the first follow-up visit. We calculated means and 95% CIs for change in pain using the KOOS and VAS_NA_, and STV between baseline and the postinjection visit. The means and CIs were calculated for those who were and those who were not OARSI responders separately. Following this, we looked at within-person change in symptoms and STV between the follow-up visit and final visit. To do this, we calculated means and 95% CIs for within-person change for all patients who had responded to treatment and separately for patients whose symptoms had relapsed within the 6-month observation window and those whose symptoms had not relapsed during the 6 months of study (the ‘persistent responders’).

To evaluate whether there was an association between change in pain and change in synovitis across all three study visits, we used fixed-effects multiple linear panel regression (generalised multiple linear regression). The two models considered used either the KOOS pain score or VAS_NA_ as the outcome variable, the STV and study visit (coded as dummy variables) as the predictor variables, and the subject ID as a panel variable. Formally written, the first model was therefore y_it_=X_it1_β+X_it__2_β+α_i_+u_it_, where y_it_=KOOS pain subscale score, and X_it1_=STV, X_it2_=study visit (coded as dummy variables), α_i_=subject and u_it_=error. In the second model tested, VAS_NA_ was the outcome variable (ie, y_it_=VAS_NA_), but was otherwise identical to the first model. Statistical analysis was undertaken using Stata V.13.1.^15^

## Results

### Subjects

A total of 127 subjects were recruited and administered a baseline steroid injection. Of these, two were excluded because their synovial fluid WCC was greater than 1.5×10^9^/L, one was withdrawn because of an adverse event (urinary tract infection), and a further subject was lost to follow-up. In total, 64 subjects (out of the 127 subjects injected) had fluid aspirated from their knee (50.4%); the median volume was 4 mL (IQR 1.5 to 8.5 mL; range 0.25–70 mL). Of these, nine had evidence of crystals (calcium pyrophosphate (5), apatite (4)). Of the 109 subjects with radiographs the majority were either grade 2 (38.5%) or grade 3 (55.1%) with the rest grade 4 (6.4%); see table 1. Of those with tibiofemoral disease, the majority were medial (76.6%) with only a small proportion (3.7%) lateral. Just over two-thirds (64.9%) had, in addition, significant patellafemoral involvement. Of the 123 who returned for their postinjection visit, three did not have CE MRI, because they experienced adverse events following the injection (see figure 1) and were therefore excluded from the analysis. The mean age of the 120 subjects in whom it was possible to assess STV at baseline and at the postinjection visit was 62.3 years (SD 10.3) and 62 (52%) were women; see table 1. Median KOOS pain score at baseline was 44.4 points (IQR 36.1–55.6), and median VAS_NA_ was 7.0 cm (IQR 5.5–7.7). The median time between baseline and follow-up scan was 8 days (IQR 7–13.5).

**Table 1:** Subject characteristics at baseline visit

Variable	Statistic (N=120)
Age (years), mean (SD)	62.3 (10.3)
Females, frequency (%)	62 (51.7)
Number of days to follow-up appointment, median (IQR)	8.0 (7.0 to 13.5)
KOOS pain subscale score (0–100)*, median (IQR)	44.4 (36.1 to 55.6)
Pain on nominated activity VAS (0–10)†, median (IQR)	7.0 (5.5 to 7.7)‡
Pain in last week VAS (0–10)†, median (IQR)	6.5 (4.7 to 7.8)‡
Maximal Kellgren–Lawrence grade in either patellofemoral or tibiofemoral compartment	–
Grade 2, frequency (%)	42 (38.5)§
Grade 3, frequency (%)	60 (55.1)§
Grade 4, frequency (%)	7 (6.4)§
Synovial tissue volume (mm^3^), median (IQR)	8177 (5743 to 13 056)¶
Synovial fluid volume (mm^3^), median (IQR)	8158 (5699 to 12 457)¶
Number of responders to injection, at follow-up visit, frequency (%)	85 (70.8)

*KOOS pain subscale is scored from 100 (no pain) to 0 (extreme pain).

†VASs are scored from 0 (no pain) to 10 (pain as bad as you can imagine).

‡Five patients and three patients neglected to complete their pain on nominated activity VAS and pain in last week VAS, respectively.

§Kellgren–Lawrence data is available only for 115 patients in total; the remaining 12 were assessed for study eligibility via MRI or arthroscopy report.

¶Nine patients’ MRI images were of insufficient quality to allow volume calculation.

KOOS, Knee Injury and Osteoarthritis Outcome Score; VAS, Visual Analogue Scale.

### Assessment of STV

Of the 120 subjects with preinjection and postinjection CE films, data on STV were available for 111 at baseline and follow-up. In nine subjects, no postsagittal image was taken or the quality of the image was considered to be poor, precluding comparison. The ICC for intraobserver reliability of the manual segmentation was excellent at 0.94 (95% CI 0.81 to 0.98). The semiquantitative and quantitative approaches to assessing STV were correlated (r_s_=0.70; 95% CI 0.64 to 0.76). The median STV at baseline was 8177 mm^3^ (IQR 5743–13 056 mm^3^).

### Outcome following steroid injection

Early outcome (baseline to postinjection visit)

Of the 120 who completed a postinjection CE MRI, 85 (71%) were defined as responders (OARSI/OMERACT) and 35 as non-responders. Following injection, five subjects did not return for a final scan—this was for personal reasons (3), loss to follow-up (1) and inability to tolerate the scan (1). Table 2 summarises the changes in symptoms and also the STV between baseline and the postinjection visit. Following injection, knee pain significantly improved on both the KOOS (+23.9 points; 95% CI 20.1 to 27.8; p<0.001) and VAS_NA_ (−3.2 cm; 95% CI −3.8 to −2.7; p<0.001). There was a reduction also in mean synovitis volume (−1071 mm^3^; 95% CI −1839 to −303; p=0.01). Those defined as responders had a greater improvement in pain than those who did not respond (between group difference in KOOS=33.1 points; 95% CI 27.2 to 39.1; p<0.001.8 and VAS_NA_=−3.5 cm; 95%CI −4.5 to −2.6; p<0.001). There was no difference in the change in pain or STV in those who had evidence of crystals in their synovial fluid at baseline and those with fluid, but without crystals (tests for interaction effects for change-by-crystal presence in linear regressions assessing change in the KOOS pain score, VAS_NA_ and STV were 0.72, 0.89 and 0.22, respectively). There was no difference in K/L grade between responders and non-responders (χ^2^ (2, N=111)=0.02; p=0.99).

**Table 2:** Synovial tissue volume and pain: baseline and follow-up visits

	Baseline visit	Follow-up visit	Difference*		Difference; non-responders†	Difference; responders†
Variable	Mean (SD)	Mean (SD)	Mean (95% CI)	p Value	Mean (95% CI)	Mean (95% CI)
Synovitis volume (mm^3^)	9935 (6470)	8864 (7802)	−1071 (−1839 to −303)	0.01	−159 (−1553 to 1235)	−1474 (−2401 to −548)
Synovial fluid volume (mm^3^)	9872 (6852)	9019 (7449)	−853 (−1615 to −90)	0.03	−416 (−1797 to 964)	−1045 (−1962 to −128)
KOOS pain subscale score‡ (0–100)	45.6 (14.6)	69.5 (19.8)	23.9 (20.1 to 27.8)	<0.001	0.5 (−4.6 to 5.5)	33.6 (30.4 to 36.8)
Pain on Nominated Activity VAS (0–10)	6.53 (1.8)	3.30 (2.7)	−3.23 (−3.8 to −2.7)	<0.001	−0.7 (−1.5 to 0.2)	−4.2 (−4.8 to −3.7)

*Within-person difference between baseline and follow-up visit.

†Responders are those who satisfied OARSI response criteria.

‡Lower KOOS scores denote worse pain.

KOOS, Knee Injury and Osteoarthritis Outcome Score; VAS, Visual Analogue Scale.

#### Late outcome (postinjection to final scan)

Of the 85 subjects who responded, five did not return for a third MRI scan. Of the 80 subjects in whom MRI was performed, 57 (71%) had a relapse within 6 months of their injection and 23 (29%) did not (the ‘persistent responders’). Table 3A, B summarises the changes in symptoms and also STV between the postinjection and relapse visit. Among those whose pain relapsed (57), there was a significant worsening of their pain including KOOS (−27.7 points; 95% CI −32.0 to −23.4; p<0.001) and VAS_NA_ (+4.4 cm; 95% CI 3.7 to 5.1; p<0.001). In addition, there was a significant increase in STV (within-person change=+1220 mm^3^; 95% CI 25 to 2414; p=0.05). Among those whose pain did not relapse (23), there was a small within-person worsening in their KOOS pain score (−10.6; 95% CI −17.3 to −3.8; p<0.001) and also VAS_NA_ (1.3; 95% CI 0.2 to 2.4; p=0.02), though there was no significant change in STV (−202 mm^3^; 95% CI −2008 to 1604; p=0.83).

**Table 3:** Synovial tissue volume and pain: baseline, follow-up and final visits, split by response type

Variable	Baseline, mean (SD)	Follow-up, mean (SD)	Final Visit, mean (SD)	Difference; Baseline to follow-up Visit	Difference; Follow-up to final Visit
				Mean (95% CI)	Mean (95% CI)
(A) Initial responders, whose pain recurred within 6 months (‘Relapsers’)					
Synovitis volume (mm^3^)	10 319 (5419)	8609 (5517)	9829 (5048)	−1710 (−2904 to −515)	1220 (25 to 2414)
Synovial fluid volume (mm^3^)	10 023 (5408)	8690 (5514)	9860 (5152)	−1334 (−2555 to −113)	1170 (−51 to 2391)
KOOS pain subscale score (0–100)	45.1 (13.6)	75.57 (14.7)	47.89 (17.1)	30.42 (26.1 to 34.7)	−27.7 (−32.0 to −23.4)
Pain on nominated activity VAS (0–10)	6.5 (1.5)	1.99 (1.8)	6.41 (2.3)	−4.5 (−5.2 to −3.8)	4.4 (3.7 to 5.1)
(B) Initial responders, whose pain did not recur within 6 months (‘Persistent responders’)					
Synovitis volume (mm^3^)	8627 (5308)	7782 (4436)	7580 (5401)	−845 (−2651 to 961)	−202 (−2008 to 1604)
Synovial fluid volume (mm^3^)	8676 (5602)	8322 (5407)	7663 (5323)	−355 (−2201 to 1491)	−658 (−2504 to 1188)
KOOS pain subscale score (0–100)	42.9 (15.9)	83.9 (12.5)	73.3 (17.7)	41.0 (34.3 to 47.8)	−10.6 (−17.3 to −3.9)
Pain on nominated activity VAS (0–10)	6.5 (1.6)	2.4 (2.2)	3.6 (2.5)	−4.1 (−5.2 to −3.0)	1.3 (0.2 to 2.4)

KOOS, Knee Injury and Osteoarthritis Outcome Score; VAS, Visual Analogue Scale.

### Correlation between symptoms and structure

**Figure 3:**
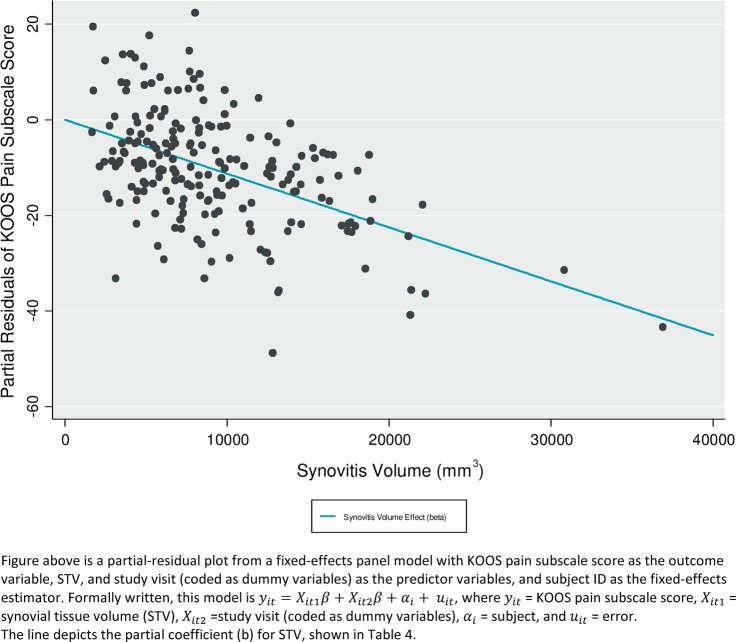
Relationship between Knee Injury and Osteoarthritis Outcome Score (KOOS) pain subscale score and synovial tissue volume (STV).

Using generalised linear modelling and adjusting for the effect of person and time (all three study visits), there was a significant association between synovitis volume and pain score (for each of KOOS and VAS_NA_, p<0.0001), see figure 3 and table 4. The increase in variance explained by the inclusion of STV in the models was, however, relatively small (change in R^2^ is 0.02 for the KOOS model and 0.04 for the VAS_NA_ model). Limiting the analyses to all initial responders (later persistent responders and those who failed) and examining pain change after initial response to later evaluation, there was a significant association between change in the level of synovitis and change in level of pain (for KOOS, p=0.01; for VAS_NA,_ p=0.04). There was no evidence of interaction between group status (persistent responder/failed) on this association.

**Table 4:** Relationship between STV and pain scores

	Effect of change in STV on outcome (mm^3^)			
Model outcome	b* (95% CI)	p Value	β	Change in R^2^ overall†
KOOS pain subscale score (0–100), mean (SD)	−1.13 (−1.87 to −0.39)	0.003	−0.28	0.02
Pain on nominated activity VAS (0–10), mean (SD)	0.17 (0.05 to 0.29)	0.006	0.32	0.04

Linear regression analysis.

*b coefficients have been scaled to reflect a change in STV of 1000 mm^3^. The models used to derive these coefficients were y_it_=X_it1_β+X_it2_β+α_i_+u_it_, where y_it_ =either KOOS pain subscale score or VAS_NA_, and X_it1_=STV, X_it2_=study visit (coded as dummy variables), α_i_=subject, and u_it_=error. Coefficients for study visit dummy variables not presented, for clarity.

†‘Change in R^2^ overall’=Difference in overall R^2^ between a fixed-effect panel model including study visit alone as the predictor and a fixed-effect panel model with study visit and STV as predictors—shows the amount of additional variance explained by adding STV into the fixed-effect regression models.

b, unstandardised coefficient; KOOS, Knee Injury and Osteoarthritis Outcome Score; STV, synovial tissue volume; VAS, Visual Analogue Scale; β, standardised coefficient.

## Discussion

In this open label study in men and women with symptomatic knee OA, there was a significant improvement in knee pain and a reduction in STV following intra-articular steroid injection, with a significant correlation between change in pain and change in STV. Among those whose pain recurred within 6 months, both pain and STV increased. A significant correlation was observed between increasing pain and increasing STV, when patient and time effects were adjusted for. Taken together, these data suggest that STV is a good candidate to be a treatment target in knee OA.

In this study, we used contrast enhancement to quantify synovial volume. Non-contrast-enhanced scans are unable to differentiate synovial tissue from synovial fluid. Consequently, the presence of synovial fluid resulted in misclassification. Our method involved manual segmentation of the outer layer of enhanced synovium and, to minimise errors of segmentation, used image analysis to demarcate the area of synovium from the surrounding lower-intensity areas including synovial fluid and cartilage. Assessment of STV is subject to errors of precision including segmentation—though formal testing suggested excellent intraobserver repeatability. Furthermore, there was good correlation between STV assessed quantitatively and semiquantitatively. Indeed, where STV was higher there was some evidence that the assessment of STV may have been more sensitive than in the semi-quantitative approach, which is constrained to a maximum score of 2 at any one of the 11 target evaluation sites (data not shown). In our study, there was no significant correlation between the aspirated synovial fluid volume and the decrease in STV between baseline and the immediate postinjection visit (Spearman's correlation coefficient R=−0.17) suggesting that STV did not incorporate free fluid in the joint. Our data suggest that STV assessed quantitatively is a reliable and valid method of assessment of synovitis in knee OA.

In our study, among those with serial MRI data at baseline and follow-up, the overall response rate for steroid injection was 71%. Of the 85 subjects who initially responded to injection, just over 25% were, based on OARSI-OMERACT criteria, persistent responders at 6 months, though with a trend towards increasing pain among this group. We used a higher dose of steroids (80 mg depomedrone) than others which may in part explain a longer-term response.^10^ We found a prolonged effect of steroids in some patients, and this was accompanied by a continuing suppression of synovitis, suggesting that a minority of patients with knee OA might be treated successfully with intermittently repeated steroid injections. This finding has important clinical implications and needs to be corroborated in other samples. Since we did not include a placebo control group in this study, we cannot rule out that these patients may have had inherently episodic disease, perhaps induced by crystals.

Steroids are widely used in the management of knee OA and it is assumed that their analgesic effect is related to their anti-inflammatory properties. Inflammation has long been recognised as a feature of knee OA, though recent studies using MRI scanning suggest that it may be more common than previously thought with up to 90% of subjects having evidence of increased STV on MRI scanning.^2^ Inflammation is likely secondary to release of cartilage degradation products into the joint and consequent triggering of the inflammatory cascade within the synovial lining layer manifesting as synovitis.

What about the impact of non-intra-articular targeted therapy on synovial tissue? In a recent open label study, 36 patients with symptomatic hand OA were given 120 mg of intramuscular methylprednisolone. There was a significant improvement in symptoms after 4 weeks and a non-significant trend for OARSI responders to have higher levels of baseline synovitis as assessed using ultrasound.^16^ In a more recent Egyptian study, 144 patients with symptomatic knee OA were randomised to receive either methotrexate of placebo. After 28 weeks there was a clinically relevant reduction in knee symptoms and also synovitis, assessed using ultrasound, in the methotrexate group compared with placebo.^17^ No attempt though was made to correlate the change in synovitis and pain in this study; however, these data support the view that synovitis may be a structural target in knee OA.

To our knowledge, our study is the largest of intra-articular therapy in knee OA, and the only one in which CEMRI was used to assess outcome and at multiple time points. There are a number of limitations to be considered. This was an open label study with subjects aware of the intervention; consequently, it is not possible to isolate the effect of the intervention on symptoms or structural outcomes as would be possible in a placebo-controlled trial. While some of the pain reduction could be due to regression to the mean or to a placebo response, it is unlikely that any of the observed structure–symptom relationships could be explained on the basis of a ‘placebo’ or contextual effect of the intervention. We note that the overall response was similar to that observed in other studies. The injections in this study were administered without imaging guidance, and it is possible that inaccuracy in placement may have contributed to a reduction in effect. While this may have affected the overall response rate, it seems unlikely that any inaccuracy in localisation of the injection to within the joint would have impacted the structural–symptom relationship that was a focus of this study.

In this study, we have shown that STV shrinks following anti-inflammatory therapy, that in most patients it rebounds after an interval period and changes in pain are linked with changes in synovitis. The correlation between change in pain and change in STV was small, supporting the view that other structural and non-structural factors play a substantial role in the occurrence of pain in knee OA. Further, the weak, although significant, relationship of synovial volume and pain change suggests that it may be a viable structural target in trials in OA. It may be challenging to show causal associations between the two parameters without large numbers in these trials. Even so, our data suggest that synovitis can be considered a candidate treatment target for anti-inflammatory therapies in knee OA which focus on their analgesic effect.

In conclusion, synovitis shrinks following steroid therapy in knee OA and its fluctuation correlates with the severity of knee pain. Synovitis should be considered a structural target for treatment in symptomatic knee OA.

## Supplementary Material

Web supplement
