# Supplementary material for: Synovial tissue volume: a treatment target in knee osteoarthritis (OA)
Source: Ann Rheum Dis. 2015 Jun 26;75(1):84–90. doi: 10.1136/annrheumdis-2014-206927 (PMC4717397; doi:10.1136/annrheumdis-2014-206927)
Supplement: Web supplement [file annrheumdis-2014-206927-s1.pdf]

Figure 1: TASK Study Patient Flow Diagram

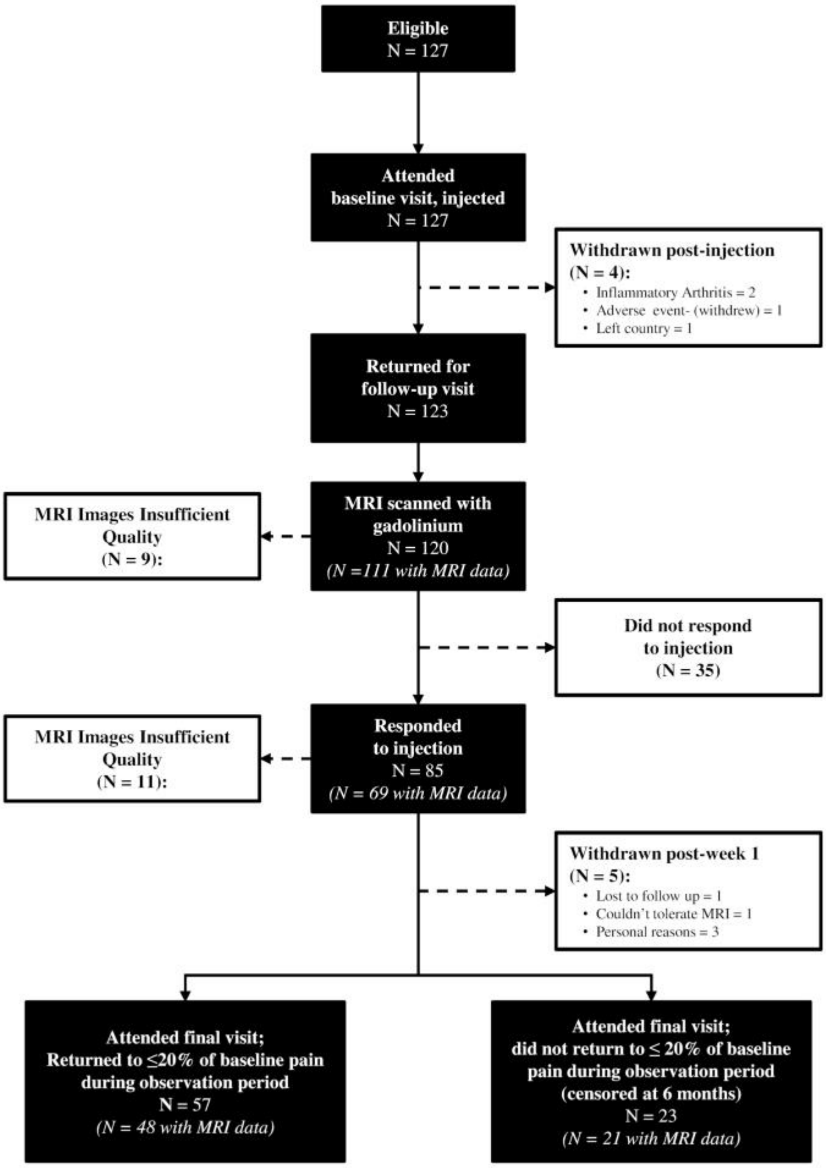

Figure 2: Gadolinium-Enhanced MRI of Patient from the TASK Study, Showing Synovial Tissue and Fluid Differentiation

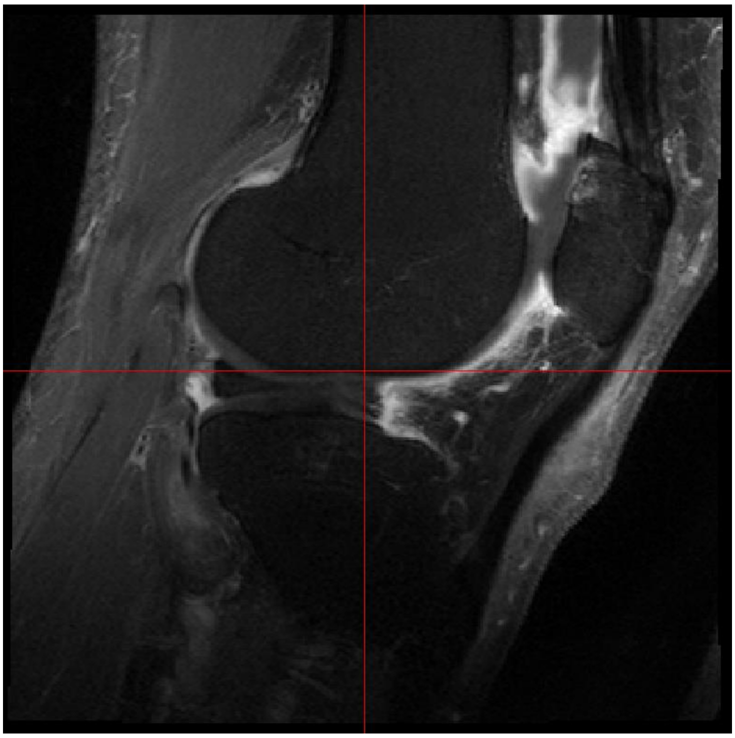

Figure 3: Relationship between KOOS Pain Subscale Score and Synovial Tissue Volume (STV).

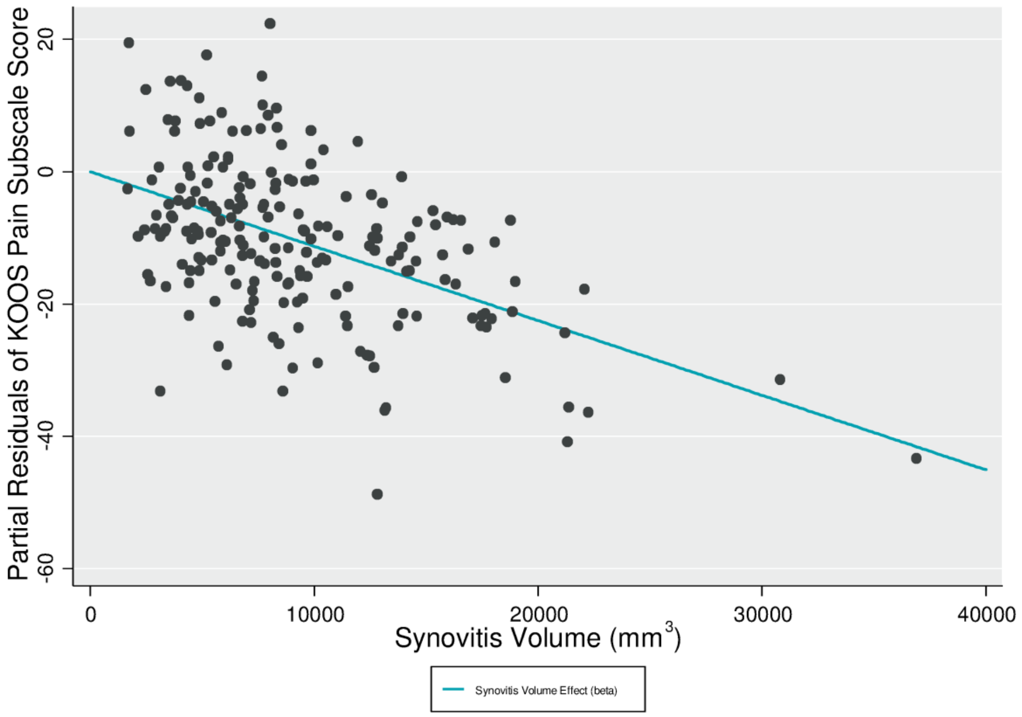

Figure above is a partial-residual plot from a fixed-effects panel model with KOOS pain subscale score as the outcome variable, STV, and study visit (coded as dummy variables) as the predictor variables, and subject ID as the fixed-effects estimator. Formally written, this model is $y_{it} = X_{it1}\beta + X_{it2}\beta + \alpha_i + u_{it}$, where y_{it} = KOOS pain subscale score, X_{it1} = synovial tissue volume (STV), X_{it2} =study visit (coded as dummy variables), α_i = subject, and u_{it} = error.
The line depicts the partial coefficient (b) for STV, shown in Table 4.
